# Super‐resolution of brain tumor MRI images based on deep learning

**DOI:** 10.1002/acm2.13758

**Published:** 2022-09-15

**Authors:** Zhiyi Zhou, Anbang Ma, Qiuting Feng, Ran Wang, Lilin Cheng, Xin Chen, Xi Yang, Keman Liao, Yifeng Miao, Yongming Qiu

**Affiliations:** ^1^ Brain Injury Center, Department of Neurosurgery Renji Hospital, School of Medicine, Shanghai Jiao Tong University Shanghai China; ^2^ Shanghai Xunshi Technology Co., Ltd. Shanghai China; ^3^ School of Electronic Information and Electrical Engineering Shanghai Jiao Tong University Shanghai China; ^4^ Department of Neurosurgery Renji Hospital, School of Medicine Shanghai Jiao Tong University Shanghai China

**Keywords:** brain tumor, generative adversarial network, magnetic resonance imaging, super‐resolution

## Abstract

**Introduction:**

To explore and evaluate the performance of MRI‐based brain tumor super‐resolution generative adversarial network (MRBT‐SR‐GAN) for improving the MRI image resolution in brain tumors.

**Methods:**

A total of 237 patients from December 2018 and April 2020 with T2‐fluid attenuated inversion recovery (FLAIR) MR images (one image per patient) were included in the present research to form the super‐resolution MR dataset. The MRBT‐SR‐GAN was modified from the enhanced super‐resolution generative adversarial networks (ESRGAN) architecture, which could effectively recover high‐resolution MRI images while retaining the quality of the images. The T2‐FLAIR images from the brain tumor segmentation (BRATS) dataset were used to evaluate the performance of MRBT‐SR‐GAN contributed to the BRATS task.

**Results:**

The super‐resolution T2‐FLAIR images yielded a 0.062 dice ratio improvement from 0.724 to 0.786 compared with the original low‐resolution T2‐FLAIR images, indicating the robustness of MRBT‐SR‐GAN in providing more substantial supervision for intensity consistency and texture recovery of the MRI images. The MRBT‐SR‐GAN was also modified and generalized to perform slice interpolation and other tasks.

**Conclusions:**

MRBT‐SR‐GAN exhibited great potential in the early detection and accurate evaluation of the recurrence and prognosis of brain tumors, which could be employed in brain tumor surgery planning and navigation. In addition, this technique renders precise radiotherapy possible. The design paradigm of the MRBT‐SR‐GAN neural network may be applied for medical image super‐resolution in other diseases with different modalities as well.

## 1 INTRODUCTION

Gliomas are the most frequently occurring primary malignant tumors of the brain and the central nervous system in adults.^1^ Accurate diagnosis and an effective therapeutic strategy are the most relevant factors on the prognosis in the event of gliomas.^2^, ^3^ Surgery and chemotherapy are the most common treatment approaches for brain tumors such as gliomas.^4^ In recent years, novel surgery methods, including gamma knife radiosurgery,^5^ and accurate radiotherapy, have resulted in a better prognosis of gliomas.^6^ Stereotactic radiotherapy (SRT) is becoming increasingly popular in treating brain tumors against whole‐brain radiotherapy, which benefits from efficient local control.^7^ A previous study conducted by our research group^8^ showed that the degree of surgical excision and precise radiotherapy were identified as the effective therapeutic factors affecting the prognosis of glioma patients. A high degree of surgical excision is also beneficial to precise radiotherapy. High‐resolution MRI images bring accuracy in detection and segmentation of tumor region, which is necessary for the clinical management of surgery and SRT.^9^

In recent years, it has been demonstrated that deep learning models are capable of accurate, efficient, and automatic segmentation of brain tumors from MRI images, which would have enormous potential value for improved diagnosis, treatment plan, surgery plan, or SRT.^10^ With the release of the brain tumor segmentation (BRATS) dataset, various deep learning models were developed to segment brain tumors. Pereira et al.^11^ proposed the automatic segmentation method based on the convolutional neural networks (CNNs) for glioma segmentation. Dong et al.^12^ proposed the UNet‐based fully connected CNNs for BRATS. UNet has now become the prominent architecture for medical image segmentation. Considering the MRI images as stacked images that could be treated as three‐dimensional volume data, the three‐dimensional convolution networks (3D‐CNNs) were proposed to improve segmentation accuracy. Baid et al.^13^ combined the 3D‐CNNs with the UNet and proposed the 3D‐UNet for achieving the higher accuracy of BRATS using the BRATS dataset. Subsequently, several modifications of the 3D‐UNet were proposed, such as cascaded 3D‐UNet,^14^ separable 3D‐UNet,^15^ and 3D‐UNet with the focal loss,^16^ which further demonstrated the potential of neural networks. Xue et al.^9^ built a brain metastases (BM) segmentation dataset comprising 1652 patients and proposed the 3D fully connected CNNs (3D‐FCN) for the segmentation and analysis of BM in the MRI images. The segmentation dice ratio reached 0.85 ± 0.08 for total tumor volume.

However, the MRI data is acquired with a finite resolution because of several limiting factors, such as the partial volume effect (PVE), hardware, imaging time, and so forth.^17^ MRI super‐resolution reconstruction is crucial to the diagnosis and segmentation of gliomas. Several algorithms have been proposed previously to deal with the problem of recovering high‐resolution MRI images.^18^ A few examples of common MRI image super‐resolution methods are diffusion tensor imaging,^19^ subpixel shifted method,^20^ inter‐slice reconstruction,^21^ self‐similarity, and image priors.^22^ In recent years, deep learning methods have demonstrated relatively better performance in MRI image super‐resolution than the traditional image processing methods. Wang et al.^17^ proposed a spare representation‐based learning method for MRI super‐resolution reconstruction, while Sert et al.^23^ proposed a residual network (ResNet)^25^ architecture based super‐resolution network for MRI reconstruction and evaluated its performance in BRATS to prove the effectiveness of super‐resolution reconstruction. However, ResNet is trained using the ImageNet dataset,^24^ which is a natural color image dataset; indicating that the input required for the super‐resolution neural network must be grayscale images with only 256 color scales rather than the original MRI image in Dicom format, which would imply loss of image information. 3D CNN methods using Dicom formatted MRI images are proposed for MRI super‐resolution.^26^, ^27^ However, in contrast to CT imaging, small slice thickness in MRI imaging usually implies a long scan time. In the MRI images of gliomas, slice thickness is generally greater than 5 mm. The performance of 3D CNNs is generally poor in the case of large slice thickness.

Wang et al.^28^ proposed the enhanced super‐resolution generative adversarial networks (ESRGAN) for single image super‐resolution (SISR), which achieved the best performance and won first place in the PRIM2018‐SR challenge.^29^ The proposed perceptual loss enables the ESRGAN network to generate realistic textures. In ESRGAN, the perceptual loss is calculated using the visual geometry group (VGG) features before the activation layer,^30^ which is fine‐tuned for material recognition.^31^ However, the medical image data collection and annotation work require professional knowledge,^32^ which makes it challenging to train an appropriate pre‐trained deep learning model to calculate the perceptual loss. Therefore, according to our knowledge, no previous study has adopted the ESRGAN architecture for medical image super‐resolution.

Intending to overcome the above‐stated challenges, the present research proposes a novel paradigm for designing super‐resolution deep neural networks for medical images. In the present research, several MRI T2‐fluid attenuated inversion recovery (FLAIR) brain tumor images were annotated and combined with the BRATS dataset to develop a new BRATS dataset. A UNet network is trained using the segmentation dataset. The UNet architecture comprises feature extraction (encoding) part and feature fusion (decoding) part.^33^ The output of the feature extraction part was utilized to calculate the perceptual loss. Moreover, long‐range identity mapping and skip connections were added to the ESRGAN network architecture. The perceptual loss and the network architecture were combined to form the MRBT‐SR‐GAN for super‐resolution of brain tumor MRI T2‐FLAIR images. In this paradigm, the specific application‐oriented medical image super‐resolution neural network could achieve the state‐of‐the‐art by means of the UNet model trained with another modality/disease.

## 2 METHODS

### 2.1 Study populations and MRI acquisitions

The institutional review board approved the present study of our hospital. All the experiments were performed in compliance with the Declaration of Helsinki. Written informed consent was obtained from each participating patient. Between December 2018 and April 2020, the data of 237 patients with gliomas were retrospectively collected. All these patients were examined in our hospital using 3.0T MRI machines with different slice thicknesses. Among the MRI images of 237 patients, 167 cases had a slice thickness of 1 mm, while the remaining cases had a slice thickness of 5 mm.

### 2.2 Dataset preparation

A total of 76 cases were selected randomly. Manual segmentation of tumor on T2‐FLAIR was performed by one neuroradiologist and one radiation oncologist. The noncommercial software itk‐SNAP (version 3.8.0; http://www.itksnap.org/pmwiki/pmwiki.php?n=Downloads.SNAP3) was employed to label the tumor region, slice by slice manually.

### 2.3 MRBT‐SR‐GAN architecture

MRBT‐SR‐GAN^28^ is a compelling image super‐resolution neural network. Ideas were borrowed from ESRGAN, and some modifications were incorporated into the architecture.

#### 2.3.1 Architecture of generator

**FIGURE 1:**
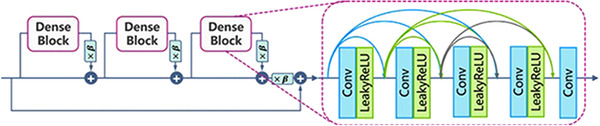
Structure of residual‐in‐residual dense block

The residual‐in‐residual dense block (RRDB) (depicted in Figure 1) proposed in ESRGAN is the basic residual block used in the generator architecture in MRBT‐SR‐GAN.^34^ The residual scaling technique and the smaller initialization technique were used to facilitate the training of the MRBT‐SR generator.^35^

**FIGURE 2:**
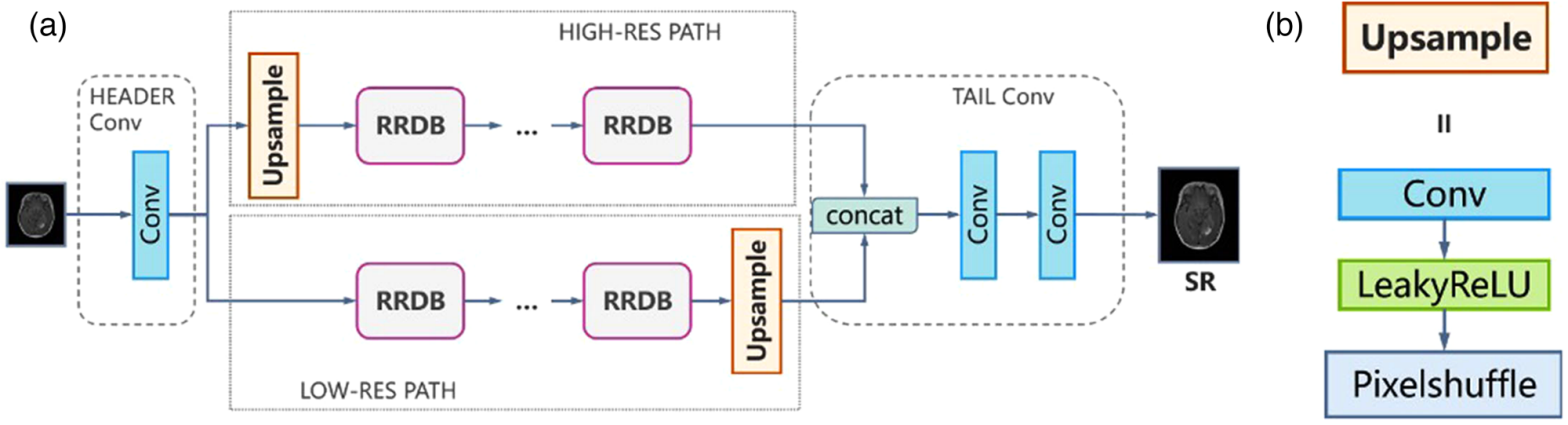
Results of super‐resolution methods: (a) structure of generator of MRI‐based brain tumor super‐resolution generative adversarial network and (b) structure of upsample block in generator

In comparison to the ESRGAN, the generator of MRBT‐SR‐GAN comprised four main components (Figure 2): (1) header convolution part, (2) high‐resolution path, (3) low‐resolution path, (4) tail convolution part. The header convolution part was used to fuse and expand the feature dimensions of the input images. The high‐resolution path was used to maintain the high‐level structure of the input images and prevent training instability. The low‐resolution path was used to obtain richer details and low‐level texture of the input images. The tail convolution part was used to fuse the high‐level and low‐level structures and generate the output, which was the final super‐resolution image with the desired channel. Each part of the four components could be modified to obtain better super‐resolution results or enable the MRBT‐SR GAN to adapt to a more significant number of applications scenes, such as slice interpolation. Further discussion on this could be obtained in the Results section.

#### 2.3.2 Perceptual loss

Johnson et al.^36^ proposed perceptual loss to achieve closer perceptual similarity. Perceptual loss is defined as the output features of a pre‐trained deep network. In SRGAN, the VGG network is used to calculate the perceptual loss.^37^ In ESRGAN, the perceptual loss is based on the features before the activation layers of a fine‐tuned VGG network for material recognition.^31^ However, the VGG network, ResNet,^25^ Inception network,^34^ and the other networks that may be used to calculate the perceptual loss are all trained using natural image datasets. In medical image processing, the image features are different for different modalities,^38^ such as computed tomography (CT) images, magnetic resonance (MR) T1 images, MR T2 fast spin echo (FSE) images, and MR T2 FLAIR images. It is difficult to identify a common network that would serve as perceptual loss for medical image super‐resolution between CT and MR images.

**FIGURE 3:**
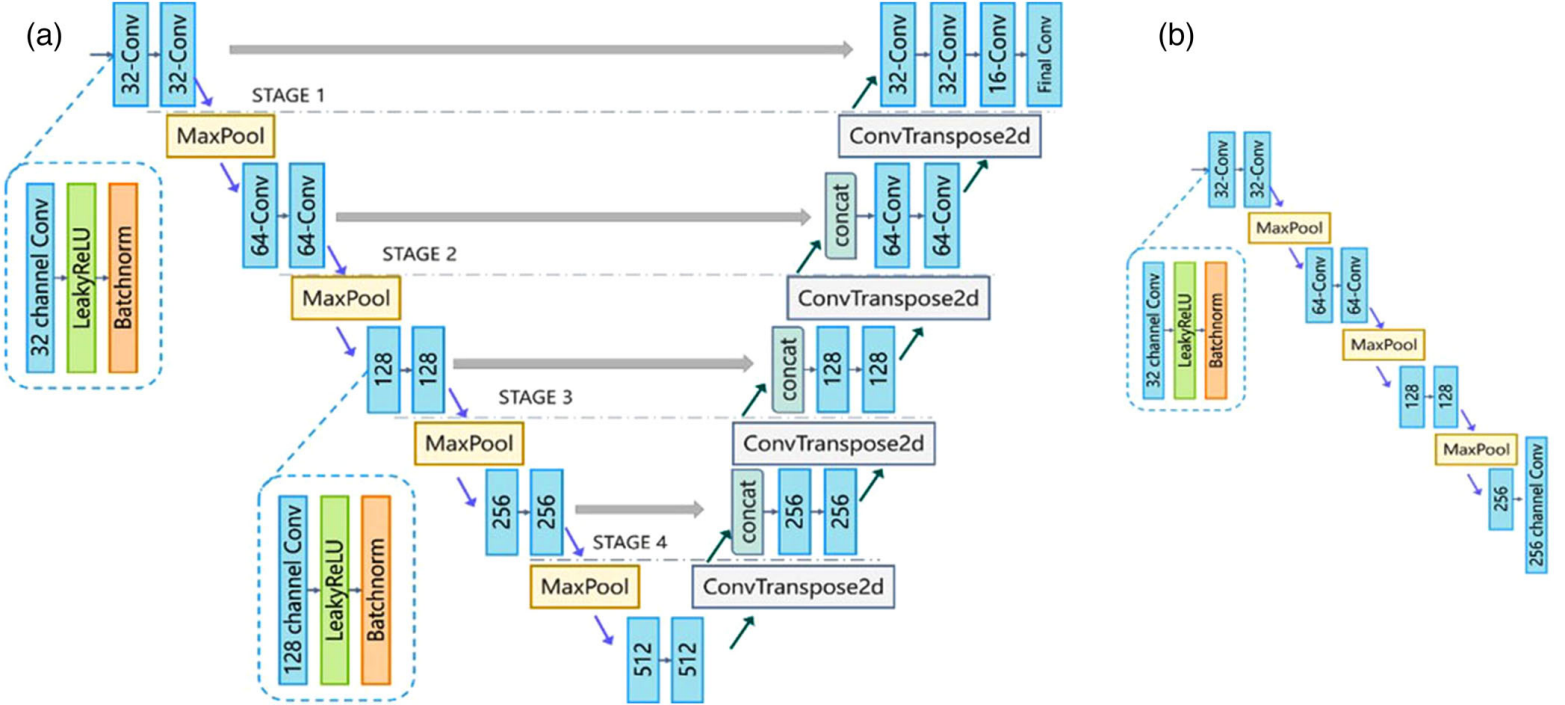
Structure of UNet: (a) structure of UNet; 32‐channel Conv implies a convolution layer with 32 output channels; final Conv block contains only the convolution layer, (b) structure of UNet used for calculating perceptual loss: 256‐channel Conv block contains only the convolution layer, and removes the LeakyReLU and Batchnorm. Figure 3b is the encoding part of Figure 3a, which is extracted for UNet as the perceptual loss of MRI‐based brain tumor super‐resolution generative adversarial network

The FCN^39^ and the derived UNet are neural network structures commonly employed in medical image segmentation tasks. The architecture of UNet comprises the encoder and decoder parts. While the encoder parts learn a hierarchy of features, decoder parts fuse the high‐level features with low‐level features and generate segmentation predictions. In MRBT‐SR‐GAN, a 2D‐UNet (Figure 3) was trained using the T2‐FLAIR images in BRATS dataset.^10^ Since the images in the BRATS dataset have a low resolution (width 240, height 240) and was preprocessed (bone subtraction, resampling), the 2D‐UNet was fine‐tuned with our manually segmented MR T2‐FLAIR images (resolution: 512 × 512) of the 76 patients who were diagnosed with gliomas. The perceptual loss was defined as the features of stage 4 in the pre‐trained UNet (Figure 3).

#### 2.3.3 Architecture of discriminator

**FIGURE 4:**
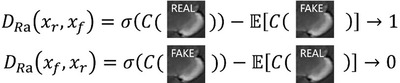
The relativistic average discriminator estimates the probability that the input data is real

The architecture of the discriminator was similar to that of ESRGAN. The relativistic average discriminator () was used to estimate the probability that a particular input image is real. The relativistic average discriminator is depicted in Figure 4.

The relativistic average discriminator () is formulated as:

The adversarial loss for the generator is in a symmetrical form, as follows:

This kind of discriminator assists in learning sharper edges and further detailed textures.^28^

**FIGURE 5:**
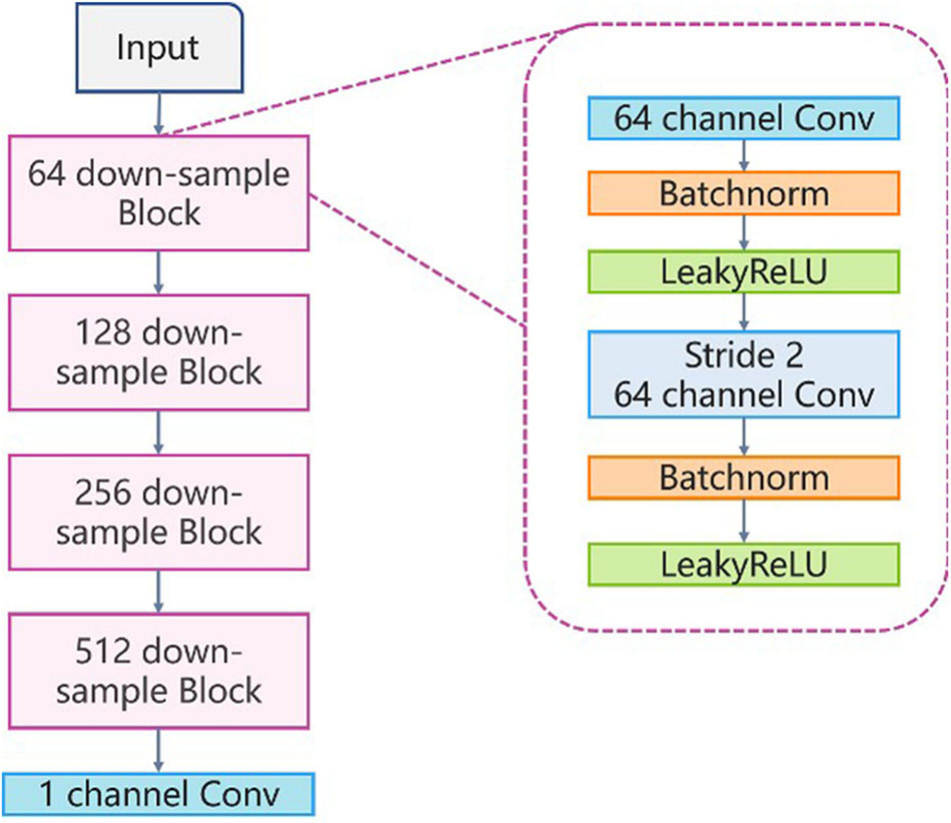
A standard GAN contains a generator and discriminator. It is the structure of discriminator of MRI‐based brain tumor super‐resolution generative adversarial network

The structure of the discriminator is a VGG‐like deep neural network (Figure 5).

Therefore, the total loss for the generator is:

where, is the content loss or pixel loss that evaluates the L1‐norm distance between the generated image and the ground‐truth high‐resolution image, and are the coefficients used for balancing the different loss terms.

### 2.4 Statistical analysis and quantitative performance measures

To quantitatively evaluate the performance of the super‐resolution of MRI T2 FLAIR images, the following three metrics were used for comparing the reconstructed images with the original ones:

Root mean square error (RMSE) which quantified the pixel intensity differences between the generated high‐resolution images and the ground‐truth high‐resolution images, using the following equation:

where, denotes the foreground regions (brain regions), and RISE denote the image intensities in the foreground regions of the generated high‐resolution images and the ground‐truth high‐resolution image, respectively. We use the skull‐stripping filter of ITK to extract the brain region. Lower RMSE means better super‐resolution performance.

Peak signal‐to‐noise ratio (PSNR), which measured the reconstruction accuracy, expressed the logarithmic decibel scale. The PSNR was calculated as follows:

Normal Range of PSNR is 20 dB to 50 dB. Higher PSNR values representing better performance.

Structural similarity index (SSIM)^40^ measured the similarity between two images. The default settings of k1 and k2 in tensorflow and pytorch deep learning framework is 0.01 and 0.03.^41^ The SSIM was calculated using the following equation:

where and denote the mean values of the generated high‐resolution images and the realistic high‐resolution images, respectively, and denote the standard deviation of and, respectively, represents the covariance of, and (L being the maximum intensity value; and). The resultant SSIM index was a decimal value within the range of −1 to 1, where 1 was reachable only in the case of two identical images. A higher SSIM value means better super‐resolution performance.

To evaluate the improved performance of automatic BRATS with the application of MRBT‐SR‐GAN, the metrics of dice ratio,^41^ specificity, and sensitivity were calculated to evaluate the accuracy of the segmentation. The definitions of these metrics are provided below.

where, and represent ground‐truth segmentation and automatic segmentation, respectively, and denotes the overlap of and. The TP score was the number of tumor pixels correctly identified as tumor pixels. The false positive (FP) score was the number of non‐tumor pixels incorrectly identified as tumor pixels. The TN score was the number of background pixels that were correctly identified as background pixels. The false‐negative (FN) score was the number of non‐background pixels incorrectly identified as background pixels.

### 2.5 Implementation details

#### 2.5.1 Data processing

Each input axial slice of an MRI T2 FLAIR image was normalized through the following steps: (1) the mean intensity value and the standard deviation of the foreground pixels were calculated, (2) the intensity value was subtracted by mean intensity value, and then divided by the standard deviation value for each pixel (including the background pixels), and (3) the high‐resolution normalized images were downsampled by a scaling factor of four using the MATLAB bicubic kernel function.

The MRI T2 FLAIR images of 100 unlabeled cases diagnosed with gliomas were used to form the training and test datasets. The training dataset comprised 70 cases (40 cases with 1 mm slice thickness and 30 cases with 5 mm slice thickness). The test dataset comprised 30 cases (10 cases with 1 mm slice thickness and 20 cases with 5 mm slice thickness). Each sample contained a pair of normalization low resolution (LR) images (128 × 128 resolution) and normalization high (H) resolution image (512 × 512 resolution).

In addition, the MRI T2 FLAIR images from the BRATS dataset were normalized to form the expended dataset. Each dataset sample contained one pair of normalization LR images (60 × 60 resolution) and non‐normalized high‐resolution image (240 × 240 resolution).

#### 2.5.2 Training settings

Our MRBT‐SR‐GAN was implemented based on the Pytorch open‐source framework on two Nvidia Titan Xp GPUs with a total memory of 24 GB. The training process involved the following two steps: (1) a PSNR‐oriented model with L1 loss was trained using the images from the expended dataset, which implied that the parameters were set to zero or one with the formulation. The model was trained through the Adam algorithm, with the initialized learning rate of 2e^−4^ was decayed by a factor of 2 every 30 epochs. The training process comprised 120 epochs in total. (2) Next, training the MRBT‐SR‐GAN model using the training dataset, the trained PSNR‐oriented model was employed to initialize the generator. The generator was trained at the following parameter settings: 1e^−1^ : 1e^−4^ : 1. The training process comprised 150 epochs, with the initialized learning rate of 1e^−4^, which was decayed by a factor of 2 every 50 epochs. The test dataset was used to evaluate the performance of the super‐resolution.

#### 2.5.3 UNet training settings

A 2D UNet based on the BRATS dataset was trained and finetuned using our manually segmented MRI T2 FLAIR images of 76 patients. The binary cross‐entropy loss and the dice loss were combined as the training loss for the 2D‐UNet. The UNet was also implemented based on Pytorch open‐source framework and trained on two Nvidia Titan Xp GPUs with a total memory of 24 GB. The training process comprised of 60 epochs in total. The initialized learning rate was 1e^−3^ and was decayed by a factor of 5 every 20 epochs. The Adam algorithm was used for the UNet. The perceptual loss is defined as the output features of stage 1 to stage 4 in the pre‐trained 2D UNet.

## 3 RESULTS

### 3.1 Super‐resolution experiment

**FIGURE 6:**
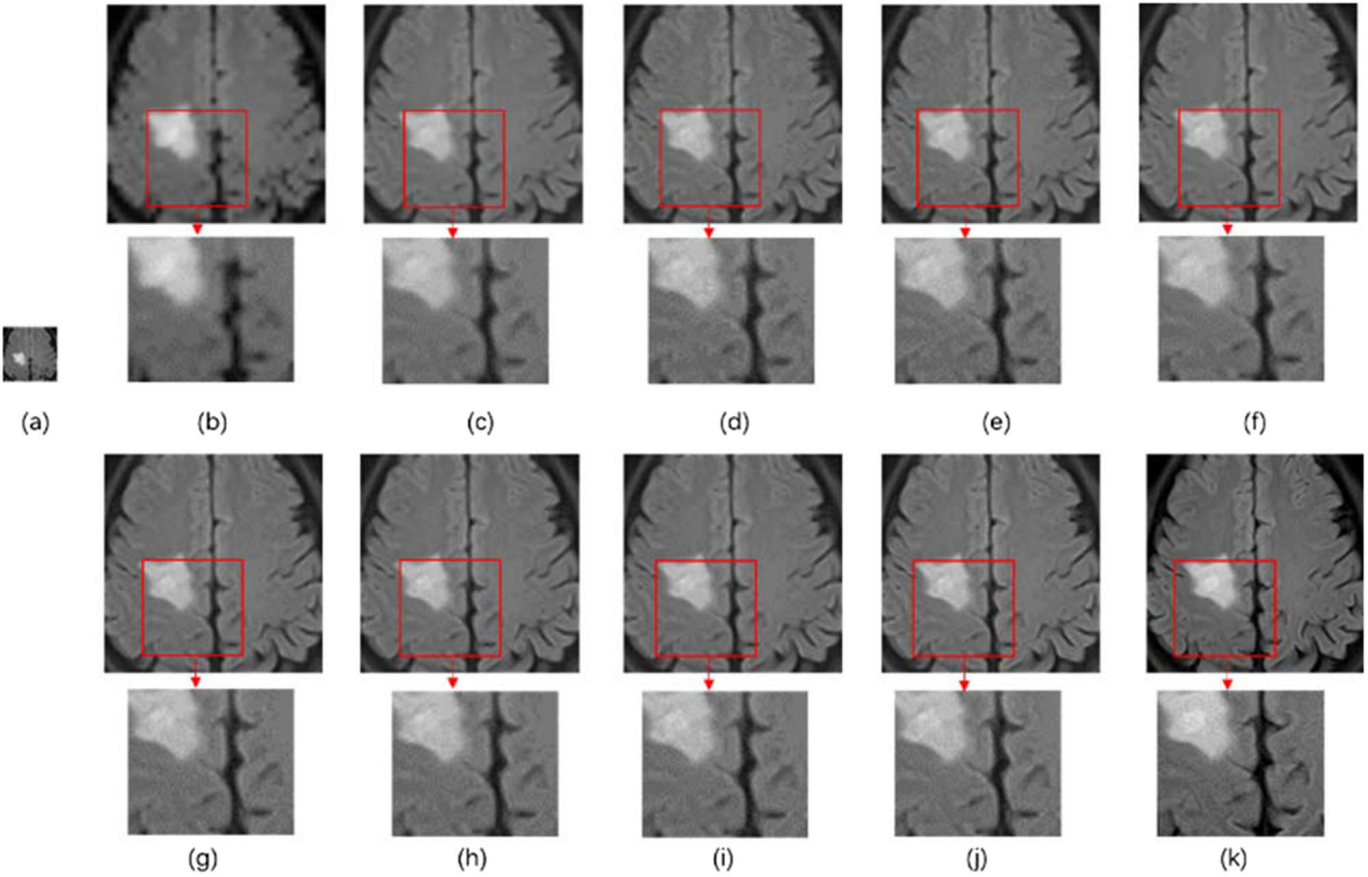
Results of super‐resolution methods: (a) 4× downsampling of the original MRI image, (b) bicubic upsampling, (c) overcomplete dictionaries, (d) enhanced super‐resolution generative adversarial networks, (e) MRI‐based brain tumor super‐resolution (MRBT‐SR) with visual geometry group perceptual loss, (f) MRBT‐SR without perceptual loss, (g) MRBT‐SR with perceptual loss (Stage 1), (h) MRBT‐SR with perceptual loss (Stage 2), (i) MRBT‐SR with perceptual loss (Stage 3), (j) MRBT‐SR with perceptual loss (Stage 4), (k) the original high‐resolution image

The super‐resolution neural network proposed in the present study was compared to the approaches by Pham et al., Chen et al., Rueda et al., and Li et al.^26^, ^27^, ^42^, ^43^ The results for the performance of super‐resolution approaches are presented in Table 1 and Figure 6. As depicted in Figure 6, the 4× downsampled image was upsampled using all of the proposed methods. The performance of MRBT‐SR‐GAN exceeded the performance of all the other methods. The MRBT architecture with the UNet features as perceptual loss presented better super‐resolution quality evaluated with RMSE, PSNR, and SSIM performance metrics. The MRBT architecture with stage 4 in the pretrained finetuned UNet as the perceptual loss has the best evaluation results.

**TABLE 1:** Comparison of bicubic, overcomplete dictionaries, MRBT‐SR‐without perceptual loss, MRBT‐SR‐with perceptual loss on benchmark data

	RMSE (Mean ± STD)	PSNR (dB) (Mean ± STD)	SSIM (Mean ± STD)
Bicubic upsampling	14.29 ± 1.16	25.13 ± 1.00	0.921 ± 0.015
Overcomplete dictionaries	11.52 ± 1.81	27.00 ± 1.95	0.969 ± 0.018
ESRGAN	11.41 ± 1.03	27.09 ± 1.11	0.972 ± 0.011
MRBT‐SR‐with VGG perceptual loss	9.91 ± 1.07	28.31 ± 1.33	0.970 ± 0.011
MRBT‐SR‐without perceptual loss	8.85 ± 0.53	29.29 ± 0.74	0.981 ± 0.007
MRBT‐SR‐with perceptual loss (Stage 1)	8.82 ± 0.998	29.32 ± 1.40	0.982 ± 0.010
MRBT‐SR‐with perceptual loss (Stage2)	8.75 ± 0. 71	29.39 ± 1.00	0.984 ± 0.010
MRBT‐SR‐with perceptual loss (Stage3)	8.73 ± 0.47	29.41 ± 0.66	0.986 ± 0.007
MRBT‐SR‐with perceptual loss (Stage4)	8.72 ± 0.49	29.42 ± 0.69	0.986 ± 0.006

Abbreviations: ESRGAN, enhanced super‐resolution generative adversarial networks; MRBT‐SR, MRI‐based brain tumor super‐resolution, PSNR, peak signal‐to‐noise ratio; RMSE, root mean square error; SSIM, structural similarity index; VGG, visual geometry group.

### 3.2 Exploration of the MRBT‐SR generator structure

**FIGURE 7:**
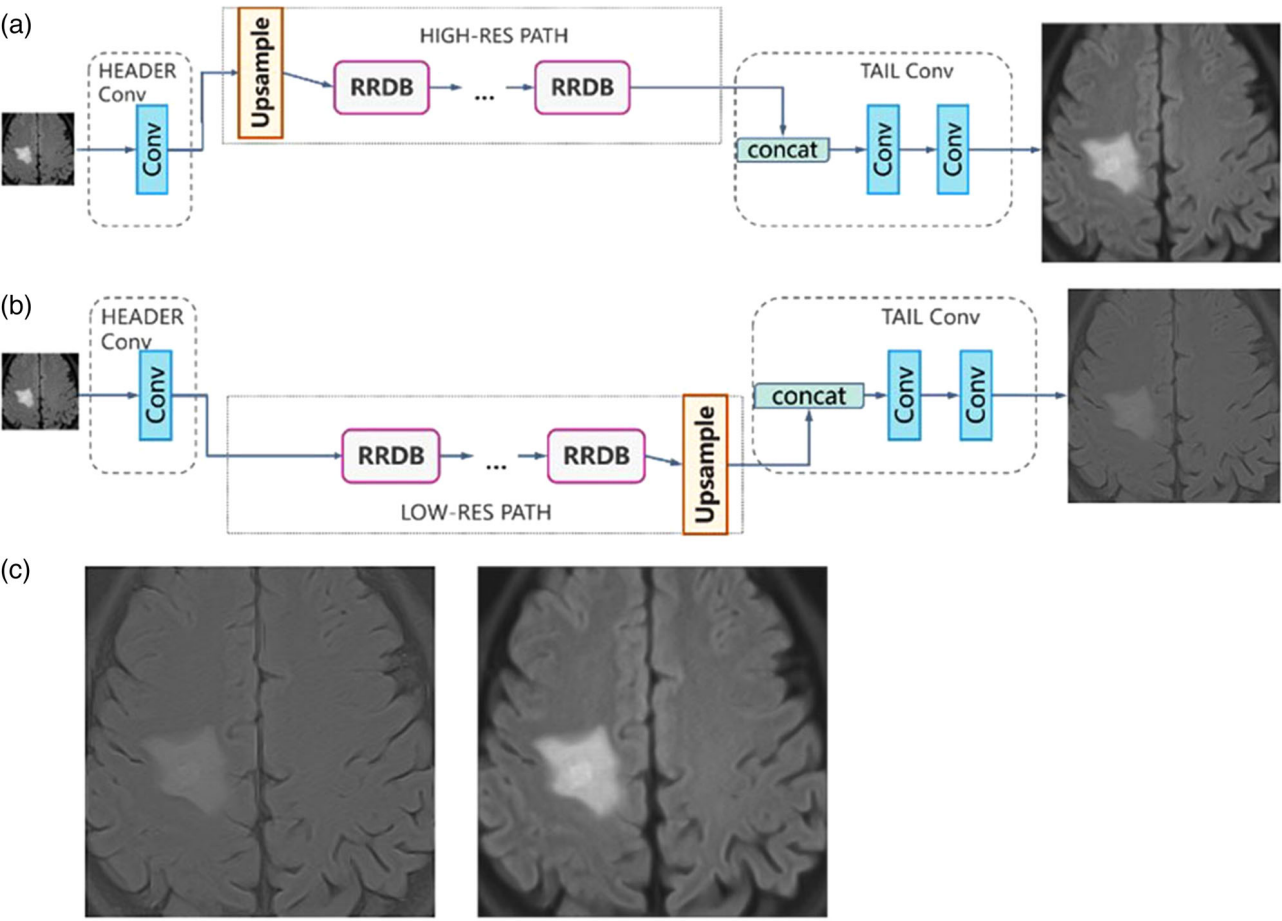
Structures of the proposed experimental neural networks and the predicted super‐resolution: (a) abandoning the low‐resolution path and retaining the high‐resolution path, (b) abandoning the high‐resolution path and retaining the low‐resolution path, (c) predicted super‐resolution results for Figure 8

The tail convolution part was used to reduce the feature channels and generate the final predicted super‐resolution results. The function of the high‐resolution path and low‐resolution path was explored in detail by abandoning another path. In the concat layer of the tail convolution part, zero tensor was used to replace the outputs of the high‐resolution path or those of the low‐resolution path. The structures of the proposed experimental neural networks and the predicted super‐resolution results are depicted in Figure 7. As depicted in Figure 7, the high‐resolution part outputs the principal part of the original image with smoother edges and textures with high fidelity. In comparison, the low‐resolution part outputs refined sharper edges and detailed textures. Combination of high‐res path and low‐res path enabled the proposed MRBT‐SR‐GAN to generate high fidelity images with detailed textures. This exploration demonstrated that the proposed MRBT‐SR‐GAN has better explanation and robustness.

### 3.3 Enhanced performance of brain tumor segmentation

A 2D‐UNet architecture depicted in Figure 3a was used to evaluate the performance of the BRATS. The experiment results are presented in Table 2. As presented in Table 2, the UNet trained with the super‐resolution dataset preprocessed by MRBT‐SR‐GAN could detect more details, particularly for the enhancing tumor (ET) and tumor core (TC) class, which implied that super‐resolution of the original images could provide more substantial supervision for brightness consistency and texture recovery. The evaluation of the three performance factors revealed that the UNet trained with the super‐resolution dataset was more accurate and robust than the UNet trained with bicubic interpolation. It was, therefore, inferred that the proposed MRBT‐SR‐GAN with features calculated by the pretrained finetuned UNet could significantly improve the BRATS accuracy.

**TABLE 2:** Comparison of improved performance contributed to brain tumor segmentation using different super‐resolution methods

	WT (Mean ± STD)	TC (Mean ± STD)	ET (Mean ± STD)
Bicubic interpolation			
Dice ratio	0.724 ± 0.11	0.611 ± 0.13	0.704 ± 0.12
Specificity	0.992 ± 0.007	0.998 ± 0.002	0.999 ± 0.001
Sensitivity	0.808 ± 0.10	0.516 ± 0.19	0.703 ± 0.13
Overcomplete dictionaries			
Dice ratio	0.758 ± 0.16	0.626 ± 0.14	0.736 ± 0.10
Specificity	0.993 ± 0.006	0.998 ± 0.001	0.999 ± 0.001
Sensitivity	0.820 ± 0.12	0.621 ± 0.16	0.765 ± 0.11
ESRGAN			
Dice ratio	0.772 ± 0.12	0.631 ± 0.11	0.745 ± 0.08
Specificity	0.994 ± 0.003	0.998 ± 0.001	0.999 ± 0.001
Sensitivity	0.832 ± 0.09	0.653 ± 0.14	0.781 ± 0.10
MRBT‐SR without perceptual loss			
Dice ratio	0.780 ± 0.10	0.635 ± 0.11	0.758 ± 0.08
Specificity	0.994 ± 0.003	0.999 ± 0.001	0.999 ± 0.001
Sensitivity	0.841 ± 0.07	0.688 ± 0.15	0.803 ± 0.09
MRBT‐SR‐GAN with perceptual loss (stage 4)			
Dice ratio	0.786 ± 0.10	0.639 ± 0.10	0.763 ± 0.07
Specificity	0.994 ± 0.003	0.999 ± 0.001	0.999 ± 0.001
Sensitivity	0.846 ± 0.06	0.709 ± 0.14	0.817 ± 0.09

Abbreviations: ESRGAN, enhanced super‐resolution generative adversarial networks; ET, enhancing tumor; MRBT‐SR, MRI‐based brain tumor super‐resolution; MRBT‐SR‐GAN, MRI‐based brain tumor super‐resolution generative adversarial network; tumor core (TC).

### 3.4 Other modifications

**FIGURE 8:**
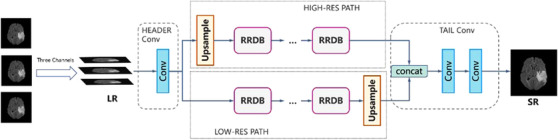
Modified structures of the generator of MRI‐based brain tumor super‐resolution generative adversarial network

The header convolution part of MRBT‐SR‐GAN was modified to take multiple consecutive slices as input, and experiments to evaluate the super‐resolution accuracy were designed. The modified structures are depicted in Figure 8. As depicted in Figure 8, three consecutive slices were utilized as the three input channels of the first convolution layer in the header convolution part. This allowed the use of five or more successive slices as input channels. The experimental results are presented in Table 3. As revealed in Table 3, the slice thickness could significantly influence the super‐resolution accuracy. The smaller the distance between the slices, the better was the super‐resolution accuracy. The structure that considered five consecutive slices as the input did not exhibit improved model performance than the structure, which considered only three consecutive slices as the input. In brief, the influence of the input slices or channels varies with the distance from the target slice. Owing to the limited number of output channels or features of the convolution layer in the header convolution part, a more significant number of input slices or channels could introduce noise in the target slice. It would ultimately reduce the accuracy of the super‐resolution.

**TABLE 3:** Experimental results for the modified structures of MRI‐based brain tumor super‐resolution generative adversarial network

	RMSE (Mean ± STD)	PSNR (dB) (Mean ± STD)	SSIM (Mean ± STD)
3 slices, slice distance:1 mm	8.34 ± 0.43	29.81 ± 0.634	0.988 ± 0.006
5 slices, slice distance:1 mm	8.39 ± 0.39	29.76 ± 0.57	0.988 ± 0.006
3 slices, slice distance:5 mm	8.76 ± 0.61	29.38 ± 0.86	0.983 ± 0.007
5 slices, slice distance:5 mm	8.85 ± 0.50	29.29 ± 0.69	0.881 ± 0.01

Abbreviations: PSNR, peak signal‐to‐noise ratio; RMSE, root mean square error; SSIM, structural similarity index.

### 3.5 Slice interpolation

**FIGURE 9:**
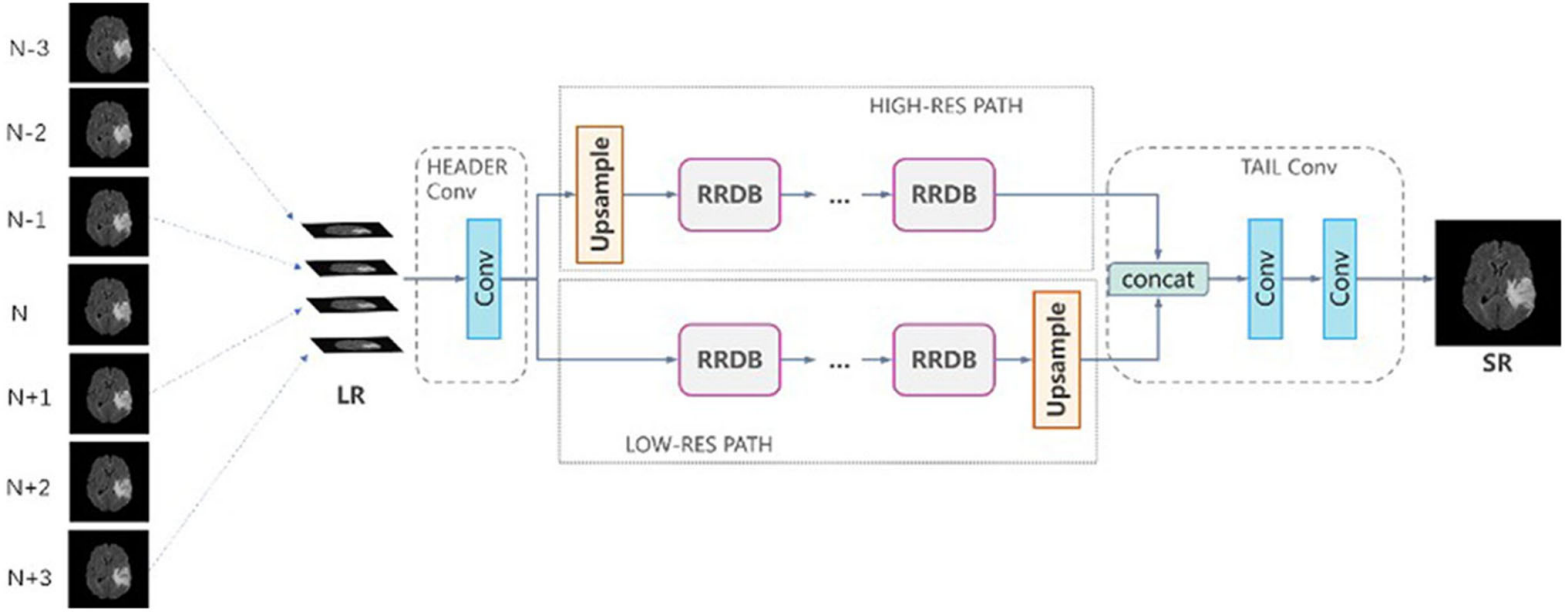
Modified structures of the generator of MRI‐based brain tumor super‐resolution generative adversarial network for slice‐interpolation. Image N represents the target slice, Image N − 1 represents the first slice above the target slice, and Image N + 1 represents the first slice below the target slice

**FIGURE 10:**
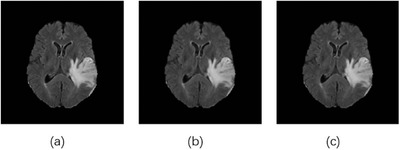
Reconstructed images generated upon slice interpolation: (a) the original image, (b) the reconstructed image by considering two nearby slices (slice distance 1 mm) as input channels, (c) the reconstructed image considering four nearby slices (slice distance 1 mm) as input channels

The MRBT‐SR‐GAN also has applications in slice‐interpolation. In the present study, the MRBT‐SR‐GAN generator structure was modified to utilize the nearby top and bottom slices of the target slice as the input channels of the first convolution layer in the header convolution part, as depicted in Figure 9, the top and bottom slices close to the target slice were utilized as input channels to train the MRBT‐SR‐GAN. The experimental results are presented in Table 4. As revealed in Table 4, the distance between the slices could significantly influence slice‐interpolation performance. The smaller the distance between the slices, the better was the slice‐interpolation performance. If the slice distance exceeds 5 mm, it will not improve the quality of the interpolated target slice. However, when the number of input channels exceeds 6, the performance of slice‐interpolation will not improve anymore, which means that structures of slices with a larger distance to the target slice will play a minor role in slice‐interpolation of the target slice. The interpolation slices could not be reconstructed from the MRI images slices with thick slice‐thickness. If the slice distance of the MRI images was less than 3 mm, it was possible to interpolate the intermediate slice or layer with high image quality. In this manner, the MRI scanning time could be reduced, and smaller residual or recurrence lesions that the original scanning images might have missed could be identified. The reconstructed images are depicted in Figure 10.

**TABLE 4:** Experimental results for slice‐interpolation using the modified structures of MRI‐based brain tumor super‐resolution generative adversarial network

	RMSE(Mean ± STD)	PSNR (dB) (Mean ± STD)	SSIM (Mean ± STD)
2 slices, slice distance:1 mm	9.40 ± 1.41	28.80 ± 1.86	0.973 ± 0.013
4 slices, slice distance:1 mm	9.29 ± 1.07	28.87 ± 1.42	0.978 ± 0.012
6 slices, slice distance:1 mm	9.30 ± 1.06	28.88 ± 1.40	0.978 ± 0.013
2 slices, slice distance:5 mm	20.73 ± 2.17	21.90 ± 1.29	0.803 ± 0.027
4 slices, slice distance:5 mm	19.25 ± 1.93	22.54 ± 1.24	0.819 ± 0.025
6 slices, slice distance:5 mm	19.51 ± 2.02	22.36 ± 1.27	0.807 ± 0.026

Abbreviations: PSNR, Peak signal‐to‐noise ratio; RMSE, root mean square error; SSIM, structural similarity index.

## 4 DISCUSSION

PVE arises in the volumetric MRI images when greater than one tissue type occurs in a voxel.^44^ The voxel intensity relies on the imaging sequence and tissue properties and the proportion of each tissue type present in the voxel. In particular, in the MRI images of brain tumors such as gliomas, the tumors are poorly circumscribed, and the edges between the tumor and the normal brain tissue are not well defined. Therefore, it is challenging to segment the brain tumor region and plan precise treatments accurately. In addition, PVE causes the early detection of cancer and accurate evaluation of the recurrence of gliomas to become difficult. MRBT‐SR‐GAN could effectively upsample the MRI images and decrease the influences of image‐blurring caused by PVE to a certain extent, which led to accurate detection and evaluation of the gliomas with small tumor regions.

Reliable BRATS is essential for accurate diagnosis and treatment planning. However, brain tumors are highly heterogeneous in terms of location, shape, and size, rendering automatic segmentation methods challenging to this day.^45^ Deep learning methods demonstrate outstanding potential in detecting and segmentation of the brain tumor regions based on MRI images. In this context, the BRATS challenge has released a large dataset of annotated MRI images of brain tumors, and this challenge has become an annual affair now. MRBT‐SR‐GAN could serve as an effective tool for improving segmentation accuracy for all the segmentation neural networks. A neural network comprising MRBT‐SR‐GAN combined with the best segmentation neural networks could greatly assist radiation oncologists in accelerating their workflow and enabling precise radiotherapy. In MRI, a thinner slice gap may introduce artifact and noise into the adjoining slices.^47^, ^48^ Although recent advances in high‐field (≥7 T) MRI have enabled the study of the fine structure of the human brain at the level of fiber bundles and cortical layers.^46^ In addition, multiple scans may reduce the slice gap and produce a high‐quality image with a small slice gap. However, high‐field MRI and the multiple scan method are expensive and time‐consuming. Therefore, they are not feasible for use in clinics. The patients with brain damage rarely cooperate with long‐term MRI examinations. In the present study, the MRBT‐SR‐GAN was modified to perform slice interpolation and obtained satisfactory results. Slice interpolation may resolve the issue of undefined brain tumor location and assist in accurately calculating the size and volume of brain tumors. The proposed MRBT‐SR‐GAN comprises five main parts: header convolution part, high‐resolution path, low‐resolution path, tail convolution part, and task‐oriented perceptual loss.

Figure 7 illustrates the functions of the high‐resolution and low‐resolution paths. Figures 8 and 9 illustrate the modifications in the header convolution part for utilizing the neighbor slices and realizing a variety of functions for further accurate super‐resolution or slice interpolation. In Table 1 and Figure 6, the influence of perceptual loss on super‐resolution accuracy is demonstrated. The five parts together formed the design paradigm for medical image super‐resolution applications. For instance, if a super‐resolution neural network for CT images is to be trained to diagnose spine fracture, one may input five consecutive slices of CT images and modify the header convolution part using 3D convolution. A UNet neural network may be trained for spine segmentation, and the output features of different stages of the UNet encoder part may be used as the perceptual loss. This would produce SPINE‐SR‐GAN architecture.

However, there are some limitations of the MRBT‐SR‐GAN models. The most important limitation is that, only T2‐FLAIR modality is included in the MRBT‐SR‐GAN, there are many MRI modalities such as T1‐weighted images, T2‐weighted images and other possible modalities could be considered and included in building the datasets, which will make the MRBT‐SR‐GAN a more adaptable and universal model in clinical.

## 5 CONCLUSION

In conclusion, our proposed MRBT‐SR‐GAN could effectively improve the resolution of MRI images while remaining high texture information, which could reduce the impact of the PVE. The improved high‐resolution MRI images could enhance the accuracy of BRATS. Modified MRBT‐SR‐GAN could do slice interpolation, which could assist in reducing MRI scanning time. Finally, the designed paradigm of MRBT‐SR‐GAN could be generalized for other medical modalities of different diseases.

## CONFLICT OF INTEREST

The authors declare no conflict of interest.

## AUTHOR CONTRIBUTIONS

Zhiyi Zhou, Yifeng Miao. and Yongming Qiu conceived this project and designed the experiments. Qiuting Feng, Anbang Ma, and Yongming Qiu designed and developed the project. Lilin Cheng, Ran Wang, Xin Chen, Xi Yang, and Keman Liao acquired data. Lilin Cheng and Xi Yang analyzed and interpreted the data. Zhiyi Zhou, Qiuting Feng, Anbang Ma, and Yifeng Miao discussed and edited the paper. All authors read and approved the final manuscript.

## ETHICS STATEMENT

The institutional review board approved the present study of Renji Hospital, Shanghai Jiaotong University School of Medicine. All the experiments were performed in compliance with the Declaration of Helsinki. Written informed consent was obtained from each participating patient.

## Data Availability

All data generated or analyzed during this study are included in this. Further enquiries can be directed to the corresponding author.
